# Green Synthesis of High Temperature Stable Anatase Titanium Dioxide Nanoparticles Using Gum Kondagogu: Characterization and Solar Driven Photocatalytic Degradation of Organic Dye

**DOI:** 10.3390/nano8121002

**Published:** 2018-12-04

**Authors:** Kothaplamoottil Sivan Saranya, Vinod Vellora Thekkae Padil, Chandra Senan, Rajendra Pilankatta, Kunjumon Saranya, Bini George, Stanisław Wacławek, Miroslav Černík

**Affiliations:** 1 Department of Chemistry, School of Physical Sciences, Central University of Kerala, Kerala 671316, India; sharanyacks@gmail.com (S.K.S.-K.S.S.); kunjumonsaranya916@gmail.com (S.K.-K.S.); 2 Institute for Nanomaterials, Advanced Technologies and Innovation (CXI), Technical University of Liberec (TUL), Studentská 1402/2, 46117 Liberec 1, Czech Republic; stanislaw.waclawek@tul.cz; 3 Centre for Water Soluble Polymers, Applied Science, Faculty of Arts, Science and Technology, Wrexham Glyndwr University, Wrexham LL11 2AW, Wales, UK; c.senan@glyndwr.ac.uk; 4 Department of Biochemistry and Molecular Biology, School of Biological Sciences, Central, University of Kerala, Kerala 671316, India; praj74@gmail.com

**Keywords:** titanium dioxide nanoparticles, green synthesis, gum kondagogu, methylene blue, photocatalysis

## Abstract

The present study reports a green and sustainable method for the synthesis of titanium dioxide (TiO_2_) nanoparticles (NPs) from titanium oxysulfate solution using Kondagogu gum (*Cochlospermum gossypium*), a carbohydrate polymer, as the NPs formation agent. The synthesized TiO_2_ NPs were categorized by techniques such as X-Ray Diffraction (XRD), Fourier transform infrared (FTIR) spectroscopy analysis, Raman spectroscopy, scanning electron microscope- Energy-dispersive X-ray spectroscopy (SEM-EDX), Transmission electron microscopy (TEM), High-resolution transmission electron microscopy (HR-TEM), UV-visible spectroscopy, Brunauer-Emmett-Teller (BET) surface area and particle size analysis. Additionally, the photocatalytic actions of TiO_2_ NPs were assessed with regard to their ability to degrade an organic dye (methylene blue) from aqueous solution in the presence of solar light. Various parameters affecting the photocatalytic activity of the TiO_2_ NPs were examined, including catalyst loading, reaction time, pH value and calcination temperature of the aforementioned particles. This green synthesis method involving TiO_2_ NPs explores the advantages of inexpensive and non-toxic precursors, the TiO_2_ NPs themselves exhibiting excellent photocatalytic activity against dye molecules.

## 1. Introduction

Nanoparticles (metal and metal oxides) of various types have been widely employed via physical and chemical methods. Although these systems have resulted in the formation of numerous extremely diverse nanostructures, many environmental toxicity issues have emerged [1]. Metal oxides have recently been widely explored because of the huge variety of structural, material and functional properties exhibited by their nanoparticles. Transition metal oxide nanoparticles have generated great scientific interest owing to their unusual properties compared with their corresponding bulk metals. Moreover, they have numerous industrial applications [2]. Metal oxide nanoparticles with disparate morphologies and sizes have been synthesized using different synthetic routes. These include hydrothermal, solvothermal, microwave, vapour deposition, spray pyrolysis and wet-chemical methods [3,4,5]. However, the usage of solvents in the chemical synthesis route adopted pose limitations for the application of NPs in medicine, pharmacy and other areas, primarily due to the toxicity caused by the solvents. Thus, there is an urgent need for the development of alternative, novel nanoparticle synthesis processes that could be exploited (at both the industrial and commercial level) in order to introduce cleaner, safer and smarter products suitable for application in communication technologies, medicine, pharmacy, agriculture and other industries. The introduction of environmentally benign approaches for designing NPs provides solutions to mounting tasks related to ecological concerns. Use of greener, safe and environmentally non-hazardous chemicals and green protocols for the synthesis of nanoparticles decreases the expense of synthesis while minimizing the utilization of harmful substances and their subsequent disposal [6].

Titanium dioxide (TiO_2_) is regarded as an extremely promising metal oxide that can perform a key role in solving the global energy crunch, as well as serving to assuage environmental concerns [7,8]. Nanoparticles of TiO_2_ have featured in numerous photovoltaic applications and in various sectors including cosmetic, pharmaceutical and skin care products. These versatile NPs can also confer whiteness to paints, plastics, papers, inks, food colorants and toothpaste while also finding usage in cancer treatment [9,10]. The synthesis of TiO_2_ NPs, which possess controlled crystal phases and morphologies that make them eminently suitable in diverse applications requiring high performance, has proven to be fundamentally challenging for the scientific community [11].

Carbohydrate polymers isolated from trees [12], are an innovative type of potentially cost-effective and biologically favorable biomaterials that display highly particular and selective characteristics towards the design of nanostructures for unique applications. They are natural biopolymers based on plant exudates and possess interesting properties e.g., reducing, stabilizing, suspending, gelling etc. [13,14,15]. Tree exudates (gums) serve as ‘green’ media and harbor numerous hydroxyl, carbonyl and carboxylic functional groups. Such functional groups can act as good chemical reductants and their presence in these gum hydrocolloid materials facilitates the formation of metal or metal oxide NPs. This is achieved in two ways: either through the reduction of metal ions or by the gum molecules behaving as a stabilizing mediator (to prevent nanoparticle agglomeration). Hence, the essential criterion for designing nanomaterials with desirable properties is fulfilled [16,17].

The Kondagogu (*Cochlospermum gossypium*, KG) is a native tree growing in the forests of India and its exudates have been categorized as being substituted rhamnogalacturonans [16]. KG is a complex and acidic polysaccharide with high solution viscosity and gelation characteristics. KG contains sugars such as arabinose, rhamnose, glucose, galactose, mannose, glucuronic acid and galacturonic acid. The structural features assigned to KG are (1→2) *β*-D-Gal *p*, (1→6) *β*-D-Gal *p*, (1→4) *β*-D-Glc*p* A, 4-*O*-Me-*α*-D-Glc*p* A and (1→2) *α*-L-Rha [18,19]. It has a high content of uronic acid and diverse functional groups (hydroxyl, acetyl, carbonyl and carboxylic) [18,20]. The morphological, physicochemical and structural analysis of this biopolymer is currently being extensively researched [18,19].

Organic dyes represent major clusters of pollutants flushed out into wastewaters from textiles and other industrialized procedures. Due to their potential injuriousness and presence in external waters, their elimination and removal has been a matter of considerable importance, since even small amounts of released dyes can discolor surface waters and impact negatively on the otherwise aesthetic and pristine surroundings [21]. The dyes altered the absorption and reflection of sunlight entering the water, which in turn affected bacterial growth and subsequently the level of biological impurities in the water [22]. Wastewater purification is discernible as one of the most serious environmental challenges of the present day. To this end, solid catalysts have been widely used in various water treatment technologies, both in processing and industry. Nanoparticles, courtesy of their small size and high surface-to-volume ratios, display high absorbing, interacting and reactive capabilities, underscoring their value in wastewater remediation [23].

Photocatalysis is an environmentally benign process involving the conversion of light energy into chemical energy under ambient conditions. The outstanding performance of solar driven photocatalytic processes in solving environmental problems has gained much attention in recent years, effluents from the textile and paint industries being the main sources of environmental organic contaminants. Thus, it is essential that the latter is degraded and converted into harmless mineral compounds. The photocatalyst can be harnessed for environmental remediation, which includes abstraction of pesticides, fungicides and fertilizers from wastewater and the eradication of organic pollutants from the air [24]. Activation of a semiconductor photocatalyst is achieved by the absorption of a photon, which results in the transfer of an electron from the valence band to the conduction band by creation of a hole (h^+^) in the valence band [25]. These photo-generated charge carriers cause redox reactions on the surface of the photocatalyst, i.e., any contaminant that is adsorbed onto the photocatalyst surface will undergo reduction or oxidation by the electron-hole pair respectively. Generally, the metal oxide photocatalyst surface acts as an active center in photocatalysis, either by the generation of OH∙ radicals (by oxidation of OH^−^ anions) or by the generation of O_2_^−^ radicals (by the reduction of O_2_.) Subsequently, these photo-generated radicals and anions react with the adsorbed organic contaminants, degrading or mineralizing them into less harmful by-products. The photocatalytic reaction can be employed to bring about the transformation of highly toxic chemicals into less noxious or non-toxic products such as CO_2_ and H_2_O [26,27]. Consequently, metal oxides can be employed as prime candidates for the effective photocatalytic degradation of environmental contaminants.

In this study, we have focused on the green synthesis of TiO_2_ NPs and their effective application in the photocatalytic degradation of a commercially used organic dye, methylene blue (MB). The influence of various solution pH values on photocatalytic efficiency was also studied.

## 2. Materials and Methods

### 2.1. Materials

Titanium oxysulfate and methylene blue were purchased from Sigma Aldrich and HiMedia Chemicals, Mumbai, India, respectively. KG samples were obtained from the Girijan Co-operative Corporation Ltd. (GCC), Hyderabad, India. All the other chemicals and solvents used were of analytical grade.

### 2.2. Fabrication of TiO_2_ Nanoparticles

Titanium dioxide NPs were synthesized using a typical procedure as described here. The procedure involved adding KG (50 mg) to 10 mL of titanium oxysulfate (0.1 M) and stirring vigorously (750 rpm) on a magnetic stirrer at 90–95 °C. Later, the product was centrifuged, washed and dried. The dried sample was calcined at 500 °C for 4 h, then pulverized and stored until further use.

### 2.3. Characterization of TiO_2_ Nanoparticles

#### 2.3.1. X-Ray Diffraction (XRD) Analysis

X-Ray Diffraction (XRD) configurations of the calcined samples were obtained with the diffraction angle range (2θ) set between 20° and 90° using a diffractometer (Rigaku Miniflex 600, Tokyo, Japan) with nickel filtered Cu Kα (λ = 1.54 Å) radiation and a liquid nitrogen cooled, germanium solid state detector. The spectral plots were compared with details obtained from Joint Committee on Powder Diffraction Standards (JCPDS), data files for analytical purposes.

#### 2.3.2. FTIR Analysis

For the Fourier transform infrared spectroscopy (FTIR) analysis, a spectrometer (Perkin-Elmer FTIR Spectrum Two, Singapore) in attenuated total reflection (ATR) mode and with the spectral range set between 4000 and 400 cm^−1^ and a resolution of 4 cm^−1^ was used.

#### 2.3.3. Raman Spectra

A Raman microscope (NICOLET DXR, Thermo Scientific, Waltham, MA, USA), equipped with an optical microscope, was used. An argon-ion (532 nm) or helium-neon (632.8 nm) laser was used for the excitation of the Raman signal with appropriate holographic notch filters to eliminate the laser line after excitation. Spectral analysis and curve fitting were performed using GRAMS/AI 8.00 Spectroscopy software (Alfasoft GmbH, Frankfurt, Germany)

#### 2.3.4. Scanning electron microscope- Energy-dispersive X-ray spectroscopy (SEM-EDX) Analysis

The elemental composition and morphology of TiO_2_ nanoparticles were determined using a scanning electron microscope (ZEISS, Ultra/Plus, Potsdam, Germany). Energy-dispersive X-ray spectroscopy (EDX) measurements were carried out using a scanning electron microscope equipped with an EDX attachment (JSM-6390 172, JEOL, Tokyo, Japan).

#### 2.3.5. TEM and High-resolution transmission electron microscopy (HR-TEM) Analysis

The nanoparticles and particle distributions determined were recorded by a transmission electron microscopy (TEM) (JEOL, JEM-2100,Tokyo, Japan). The samples were prepared on standard copper TEM grids covered with thin carbon foil. Drops of TiO_2_ nanoparticles were dispersed in 1 mL of isopropanol using ultrasound for 10 min. and a drop of the resulting solution was gently spread onto the upper surface of the carbon covered copper TEM grid.

#### 2.3.6. Particle Size Analysis

Nanoparticle size distributions were measured by centrifugal particle sedimentation (CPS) using the Disc Centrifuge technique (DC24000UHR, CPS Instruments Inc., Prairieville, LA, USA).

#### 2.3.7. BET Surface Area

The surface area of the TiO_2_ NPs was analyzed using the Brunauer–Emmett–Teller (BET) technique (Autosorb iQ, Quantachrome Instruments, Boynton Beach, FL, USA).

#### 2.3.8. Thermal Stability

Thermal properties of KG and green-synthesized TiO_2_ nanoparticles were studied using thermogravimetric analysis (TGA) by means of a Perkin Elmer STA 6000 Thermal Analyzer (Singapore) instrument.

#### 2.3.9. Optical Properties

Optical properties were determined using an ultraviolet (UV)-visible spectrophotometer (Perkin Elmer Lambda 35, Singapore) over the spectral region 200–800 nm.

### 2.4. Photocatalytic Degradation of Methylene Blue

The photocatalytic activity of green-synthesized TiO_2_ nanoparticles was scrutinized by the degradation of MB under sunlight. Photocatalytic activity in the presence of sunlight was determined under direct normal sunlight at an intensity of 100,000 Lux and the solar intensity measurement was carried out throughout the experiment at different time intervals. The degradation of dye was examined by collecting 5 mL aliquots from the reaction mixture at different intervals of time. In addition, these aliquots were centrifuged at 7000 rpm for 15 min. Photocatalytic degradation of the dye was monitored by measuring the absorbance spectra of the supernatants using a UV−visible spectrophotometer over the wavelength range 200–800 nm.

#### 2.4.1. Photocatalytic Studies Based on Catalyst Concentration

In order to study the effect of catalyst loading on solar light driven photocatalysis of methylene blue, different amounts (1–15 mg) of green-synthesized titanium dioxide nanoparticles calcined at 500 °C were added to 50 mL of dye solution. The reaction suspension was mixed thoroughly using a magnetic stirrer for 90 min in the presence of sunlight. The photocatalytic degradation of the dye was monitored by measuring the absorbance of the solution at regular intervals using a UV-visible spectrophotometer.

#### 2.4.2. Photocatalytic Studies Based on Time

In a typical experiment, 50 mL of dye solution (1.0 × 10^−5^ M) was mixed with 10 mg of TiO_2_ nanoparticles. The mixture was stirred continuously at 600 rpm in the presence of sunlight. The rate of degradation was monitored by measuring the absorbance of the solution (over the 200 to 800 nm wavelength range) with a UV-visible spectrophotometer by removing and monitoring 5 mL aliquots at defined time intervals. The process was continued for 90 min.

#### 2.4.3. Photocatalytic Studies Based on pH

The influence of pH on solar driven photocatalytic activity of the catalyst was studied by conducting the experiment at different pH values (4–9) of dye solution. Green-synthesized titanium dioxide nanoparticles (10 mg) calcined at 500 °C were added to 50 mL of the dye solution having different pH values. The resulting suspension was thoroughly mixed using a magnetic stirrer for 90 min in the presence of sunlight and the absorbance of the solution measured with a UV-visible spectrophotometer.

#### 2.4.4. Photocatalytic Studies Based on Temperature

The green-synthesized TiO_2_ nanoparticles were calcined at different temperatures (500–900 °C) and each sample (10 mg) was added to 50 mL of dye solution. The resulting suspension was thoroughly mixed using a magnetic stirrer for 90 min, in the presence of sunlight. The absorbance of the solution was measured with a UV-visible spectrophotometer.

## 3. Results and Discussion

### 3.1. Mechanism of TiO_2_ NPs Formation Via Green Synthesis

Natural tree based carbohydrate polymers—an environmentally benign medium—contain extensive numbers of hydroxyl, carbonyl and carboxylic groups which can act as good chemical reductants. The presence of these functional groups in the gum hydrocolloid material facilitates the formation of metal nanoparticles. When a metal oxide precursor is introduced into a well dissolved KG homogeneous solution or hydrogel, the polyhydroxylated macromolecules inherent in the gum matrix are able to absorb metal cations. For example, the Ti^3+^ ion could be chelated with KG by means of –OH and –COOH groups. The sequestration of cations [M*^n^*^+^] or hydroxylated cations [M(OH)]*^m^*^+^ that can undergo nucleation or growth processes is accelerated by the highly reactive hydroxyl groups present in KG. The last step in the TiO_2_ nanoparticle synthesis is the calcination process at 500 °C which removes the gum template via combustion of their organic scaffold, giving rise to the formation of dispersed oxides [11,28]. While XRD and Raman spectroscopy confirmed the formation of anatase as the only distinguished mineral phase; FTIR, TGA and EDX collectively proved that no organic compounds (polysaccharides, hydroxyl, carboxylate and other oxidic groups) were present in the final nanoparticles.

### 3.2. XRD Analysis

**Figure 1:**
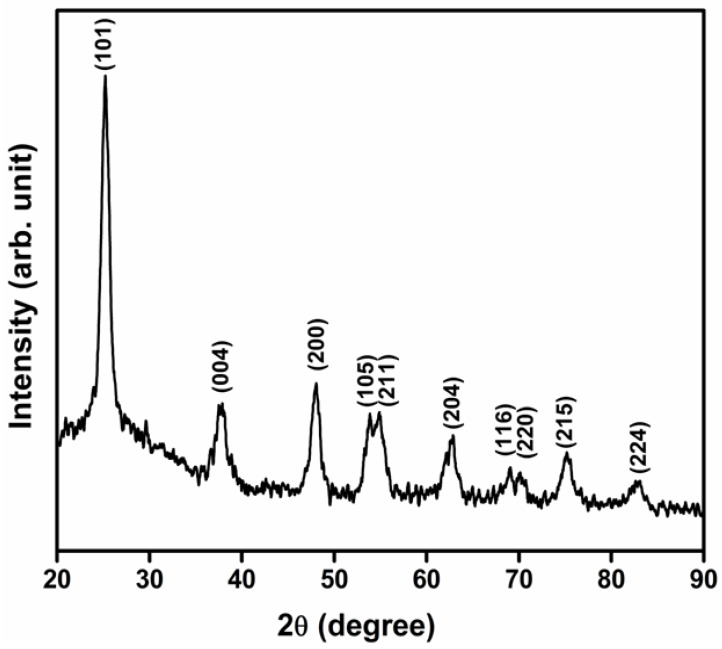
X-ray diffraction analysis (XRD) pattern of titanium dioxide (TiO_2_) nanoparticles calcined at 500 °C.

The XRD pattern of TiO_2_ NPs calcined at 500 °C showed a dominant peak at the 2*θ* value of 25.25° (Figure 1). This matches the (101) crystallographic plane of the TiO_2_ anatase structure, indicating that the crystal composition is predominantly anatase. The other characteristic diffraction peaks were at the following 2*θ* values: 37.75, 48.02, 53.86, 54.95, 62.63, 68.88, 70.28, 75.13 and 82.60°. These values correspond to (004), (200), (105), (211), (204), (116), (220), (215) and (224) crystallographic planes of anatase, respectively (JCPDS No. 01-071-1166), thus confirming the formation of TiO_2_ nanoparticles in the anatase phase [29]. The average crystallite size of the NPs [i.e., the mean size of the ordered (crystalline) domains, which may be smaller or equal to the grain size] was calculated using the Scherrer formula [*d* = 0.89λ/βcos*θ*] to be 12.58 nm. The crystallinity and higher purity of prepared TiO_2_ NPs in the anatase form was validated by the presence of sharp peaks while the absence of peaks represented other crystallite forms of TiO_2_ [30,31,32].

**Figure 2:**
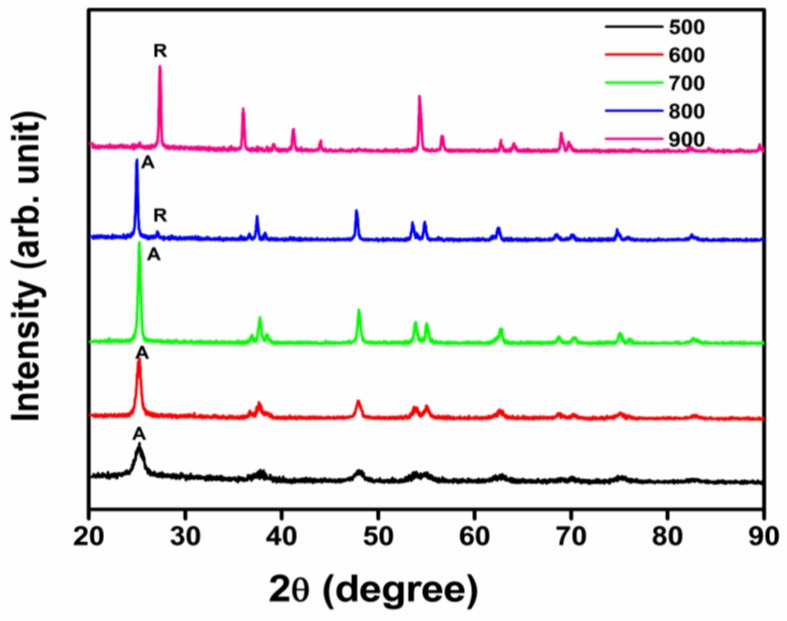
XRD pattern of TiO_2_ nanoparticles calcined at different temperatures (R denotes rutile and A denotes anatase phases of TiO_2_ nanoparticles).

XRD patterns of TiO_2_ nanocatalysts calcined at various temperatures are shown in Figure 2. When the calcination temperatures did not exceed 700 °C, anatase was the only phase, with Joint Committee on Powder Diffraction Standards (JCPDS) No. 01-071-1166, while phase transformation of TiO_2_ was observed at 800 °C. The rutile phase appeared in the 800 °C sample and became the dominant phase in the 900 °C sample with peaks at 2*θ* = 27.4°(110), 36.04°(101), 41.2°(111), 54.3°(211) (JCPDS no. 01-073-2224). Formation of the rutile phase of TiO_2_ is normally observed above 600 °C with a complete transformation to the rutile form occurring at 800 °C [33]. The present XRD patterns illustrate that the anatase to rutile phase transformation of the synthesized TiO_2_ first took place at 800 °C and was almost completed at 900 °C, thus revealing the formation of a high temperature, stable anatase phase of TiO_2_ nanocatalysts through the green synthesis method adopted.

While anatase and rutile are both mineral forms of titanium dioxide (possessing tetragonal crystal systems), in terms of optical activity, anatase is optically negative whereas rutile is positive. Furthermore, the luster exhibited by anatase is more strongly adamantine or metallic-adamantine than that shown by rutile.

### 3.3. Fourier Transform Infrared Spectroscopy Analysis

**Figure 3:**
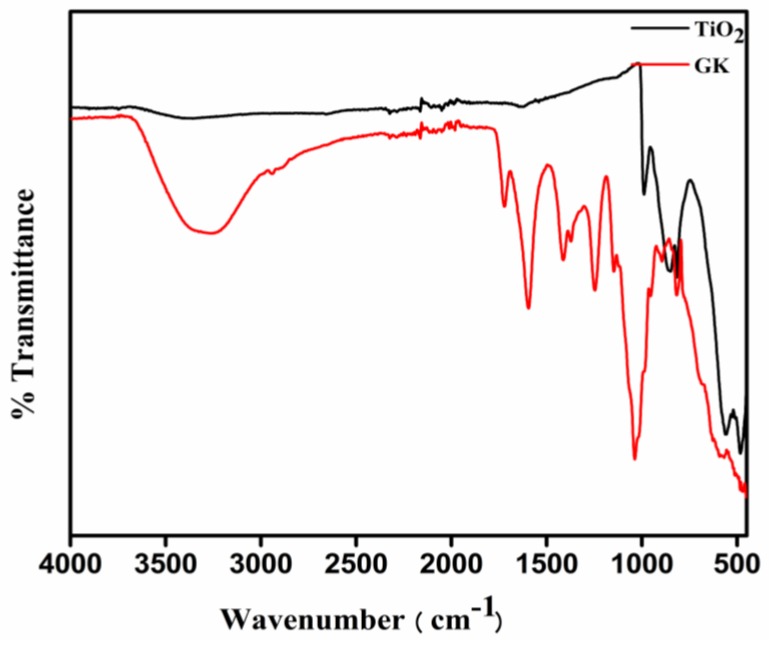
Fourier transform infrared spectroscopy (FTIR) spectra of Kondagogu (KG) and TiO_2_ nanoparticles.

FTIR spectroscopy was employed to identify different functional groups present in KG and on the surface of the formed NPs (Figure 3). The major stretching frequencies in the spectrum for KG were observed at 3368, 1719, 1609, 1417, 1247, 1145 and 1035 cm^−1^. The band observed at 3368 cm^−1^ suggests the presence of hydroxyl groups while those noted at 1719 cm^−1^ and 1609 cm^−1^ were ascribed to carbonyl stretching vibrations and the asymmetric stretching of carboxylate, respectively. The band seen at 1417 cm^−1^ was due to the symmetrical stretching of the carboxylate group present in the gum’s uronic acid. The presence of an acetyl group was inferred by the band appearing at 1247 cm^−1^ while the bands registered at 1145 and 1035 cm^−1^ were indicative of C–O stretching vibrations of ether and alcohol groups, respectively [34]. The absence of these characteristic peaks in the FTIR spectra of TiO_2_ nanoparticles promoted by KG may be a consequence of the higher purity of prepared nanoparticles in anatase crystal formations on calcination at 500 °C. The characteristic signal for TiO_2_ nanoparticles observed below 1000 cm^−1^ in the FTIR spectra was due to Ti-O-Ti vibrations [35].

### 3.4. Raman Spectroscopy

**Figure 4:**
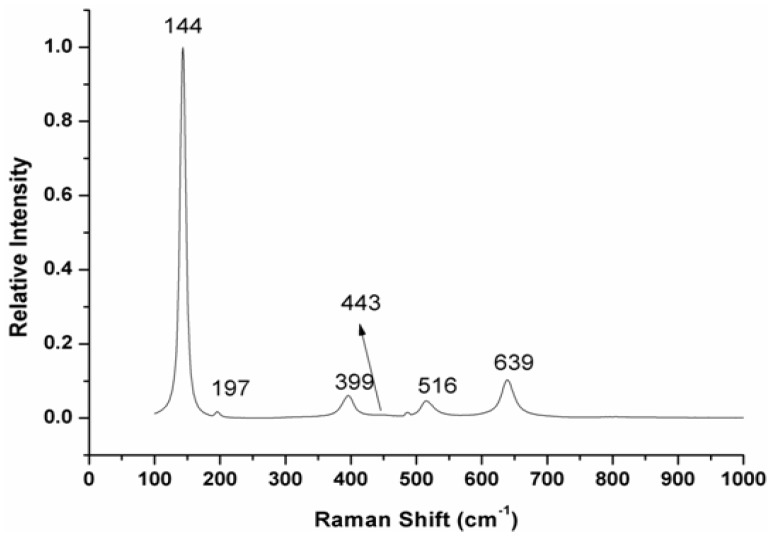
Raman spectrum of TiO_2_ nanoparticles.

To identify and quantify both the amorphous and crystalline TiO_2_ phases, Raman spectroscopy was employed. Figure 4 reveals that TiO_2_ anatase displays Raman bands at 639, 516, 399 and 197 cm^−1^, as well as a precise sharp and intense peak at 144 cm^−1^. According to the literature, the anatase phase of TiO_2_ has six Raman active modes, namely *A_1g_* + 2*B_1g_* + 3*E_g_*, determined by group analysis *D_4h_*(I4l/amd) [36], and known in the first-order Raman spectrum of single crystal TiO_2_ at 144 cm^−1^ (*E_g_*), 197 cm^−1^ (*E_g_*), 399 cm^−1^ (*B_1g_*), 516 cm^−1^ (*A_1g_* + *B_1g_*) and 639 cm^−1^ (*E_g_*) [37].

### 3.5. Scanning Electron Microscopy (SEM)

**Figure 5:**
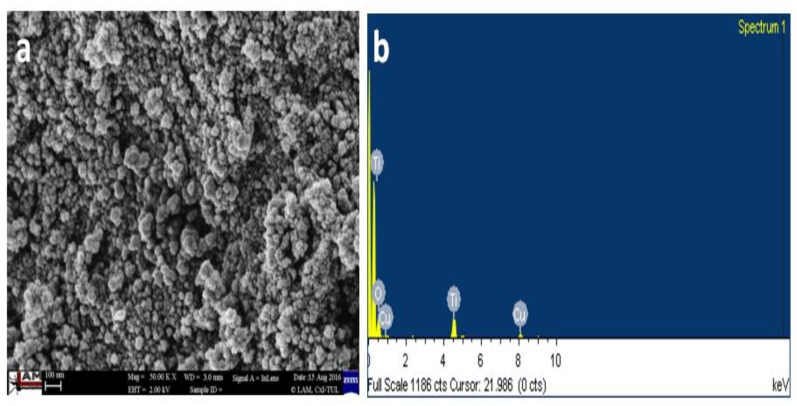
Typical (**a**) scanning electron microscopy (SEM) and (**b**) EDX (Energy Dispersive X-Ray) analysis of TiO_2_ nanoparticles.

The SEM micrograph of TiO_2_ nanoparticles is shown in Figure 5a, and the resulting nanoparticles were observed to have a spherical morphology. The chemical composition, analyzed using EDX spectra, confirmed the presence of Ti and O (Figure 5b). Since the sample was coated on a copper grid, peaks corresponding to Cu were also visible in Figure 5b.

### 3.6. TEM and HR-TEM Analysis

**Figure 6:**
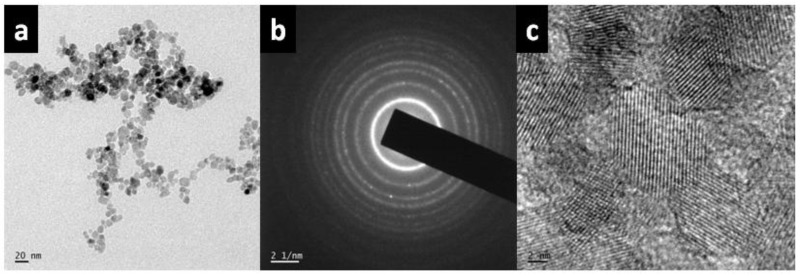
(**a**) TEM (Transmission Electron Microscopy) image, (**b**) SAED pattern and (**c**) HR-TEM (High Resolution TEM) micrograph of TiO_2_ nanoparticles.

Both particle size and morphology of TiO_2_ were confirmed by TEM analysis (Figure 6a) which revealed that the particles are monodisperse and spherical in shape. The sizes of particles were in the 8–13 nm range and the selected area electron diffraction (SAED) pattern indicated that the TiO_2_ nanoparticles possessed good crystallinity (Figure 6b). HR-TEM observations (Figure 6c) suggest that the TiO_2_ nanoparticles have a perfect lattice structure. The particle sizes determined by the TEM image were similar to the reported values obtained by applying the Scherrer equation to the XRD patterns (12.58 nm). The aforementioned equation correlates the size of sub-micrometre particles or crystallites (in a solid) to the broadening of a peak in a diffraction pattern. It is employed for the determination of the size of particles of crystals in the form of powders.

### 3.7. Particle Size & BET Analysis

**Figure 7:**
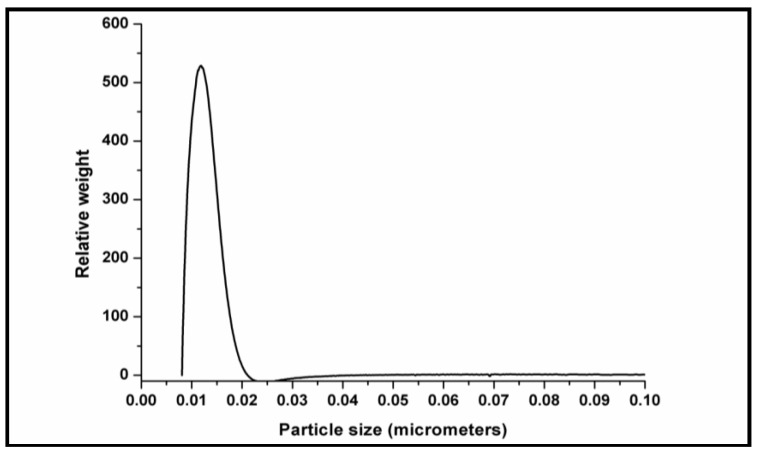
Particle size distributions of TiO_2_ nanoparticles as determined by the centrifugal particle sedimentation (CPS) method.

The TiO_2_ particle size distribution analyzed by CPS determined the mean size to be 11.2 ± 0.2 nm (Figure 7). Evidently, the TiO_2_ nanoparticles appeared to be more stable in the current study involving green synthesis and the particle size measurements corresponded very well with the TEM analytical data (Figure 6). The specific surface area of TiO_2_ was determined from the isotherms to be 42.6 m^2^/g based on the BET analysis. 

### 3.8. Thermal Studies

**Figure 8:**
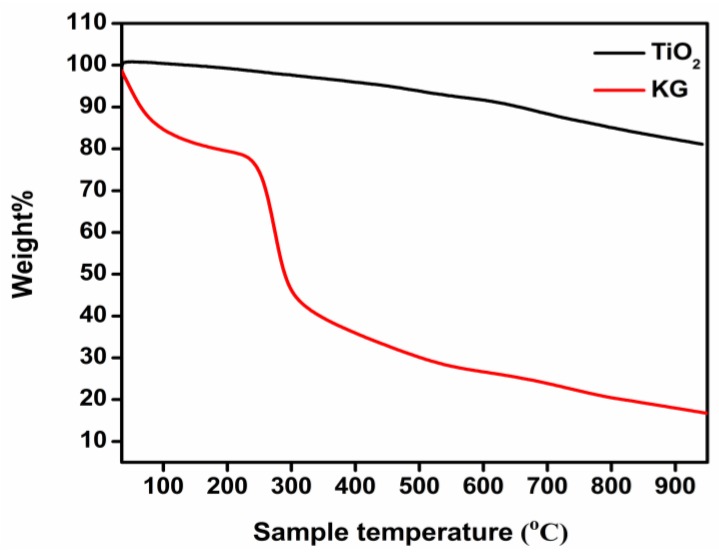
Thermogravimetric analysis (TGA) curves of KG and green-synthesized TiO_2_ nanoparticles.

Thermal properties of KG and green-synthesized TiO_2_ nanoparticles were studied using thermogravimetric analysis. Depicted in Figure 8 are the TGA curves of KG and TiO_2_ nanoparticles heated from 35 °C to 950 °C. In the case of KG, there were two major weight loss events. The first occurrence (observed between 35 °C and 111 °C) of approximately 17% probably represented the loss of adsorbed water – as hydrogen bonded water – from the polysaccharide structure. The second weight loss event of roughly 33% was very significant and was discerned between 232 and 309 °C. It was ascribed to decomposition of the polysaccharide. A third, far smaller weight loss instance was registered at 590 °C, possibly due to the conversion of the remaining polymer to carbon residue [20].

The TGA curve of TiO_2_ (Figure 7b, red curve) did not indicate any weight loss up to 766 °C, a reflection of its high thermal stability, probably due to four hours of calcination at 500 °C. There were small linear weight reductions detected in the range of 766–911 °C, possibly caused by the loss of residual carbon from the gum matrix.

### 3.9. Optical Properties

The UV-visible absorption spectrum of biosynthesized TiO_2_ nanoparticles is shown in Figure 8. The observed absorption spectrum matches those obtained with TiO_2_ nanoparticles produced by chemical methods, displaying a broad absorption band in the UV region, up to 380 nm [38].

**Figure 9:**
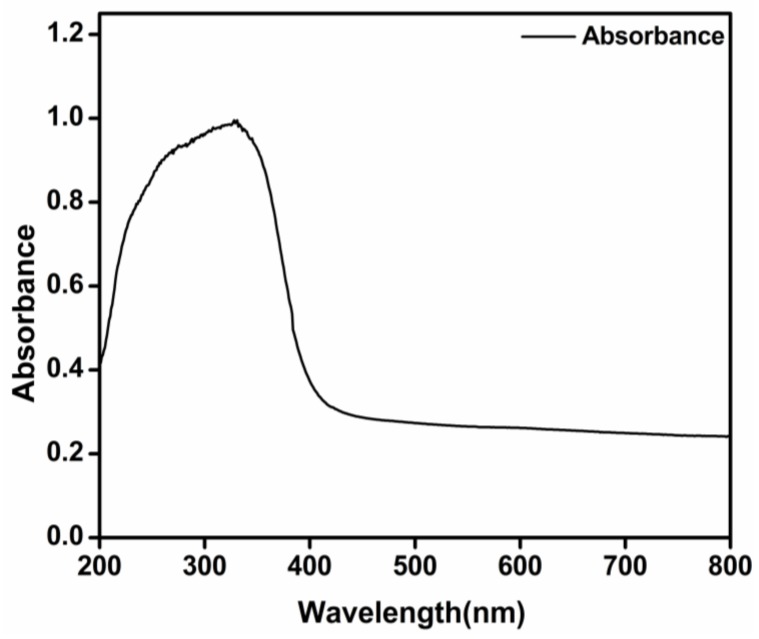
Ultraviolet (UV)-visible absorption spectrum of green-synthesized TiO_2_ nanoparticles.

**Figure 10:**
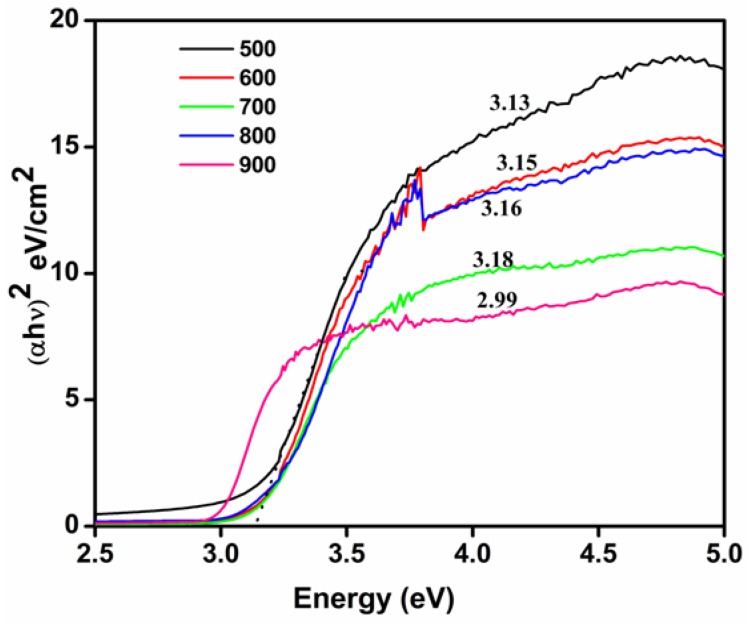
UV-Tauc plot of TiO_2_ nanoparticles calcined at different temperatures.

The UV-visible spectrum was utilized to deduce the optical absorption properties of green-synthesized TiO_2_ nanoparticles (Figure 9). The band gap energy of green-synthesized TiO_2_ nanomaterials was found to be 3.13 eV from the Tauc plot used to determine the optical bandgap in semiconductors as shown in Figure 10.

**Figure 11:**
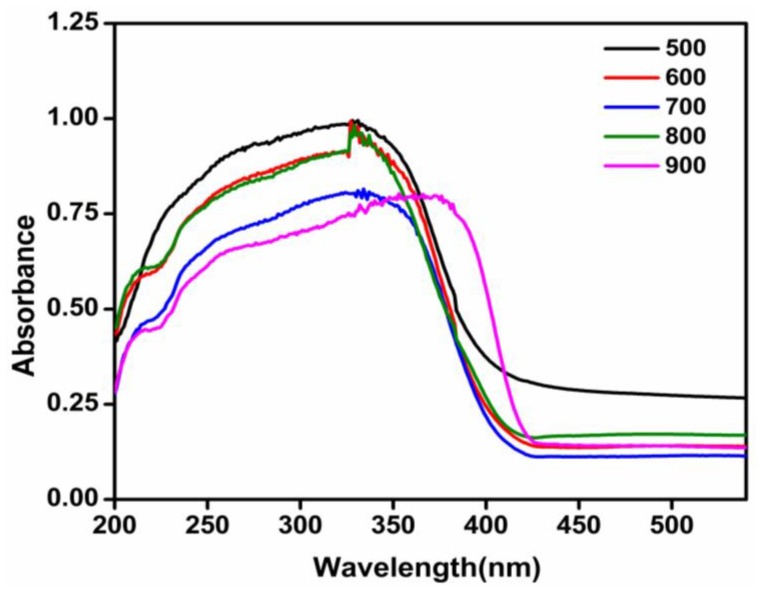
UV-Visible spectra of TiO_2_ nanoparticles calcined at different temperatures.

After the bulk, TiO_2_ had a band gap energy of 3.2 eV; the marginal reduction of 0.07 eV in this parameter being attributed to particle size dependence [39]. The absorbance spectra and the corresponding Tauc plot of TiO_2_ nanoparticles calcined at different temperatures were as given in Figure 10 and Figure 11, respectively.

The calculated band gap of TiO_2_ nanoparticles, calcined at different temperatures was as shown in Table 1. It is also evident from the UV data (Table 1) that the band gap increased with the elevation of calcination temperatures from 500 to 800 °C, before decreasing gradually at 900 °C. This variation can be expected and is plausible, given that with an increase in the TiO_2_ calcination temperature, the anatase phase became endowed with good crystal characteristics. Furthermore, the decrease in band gap at 900 °C was mainly attributed to the complete formation of the rutile phase.

### 3.10. Photocatalytic Activity

**Scheme 1:**
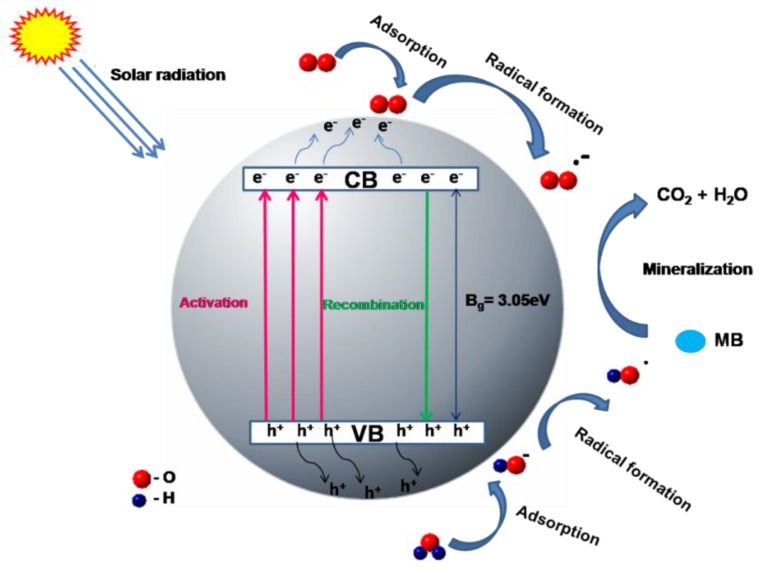
Photocatalysis mechanism of TiO_2._

The photocatalytic activity of green-synthesized TiO_2_ NPs was demonstrated by using an organic dye (methylene blue) under solar light, the dye degradation being initially identified by color change (Scheme 1). Additionally, we monitored the intensity of solar light throughout our experiment and confirmed it was in the range 100,000 Lux (see supplementary information, Figure S1). Furthermore, solar UV-radiation, as a function of time, and the UV intensity were measured using a Lux meter and UV filtering goggles. This information has been given in the supplementary document (Figures S2 and S3, respectively). The UV intensity from solar light was ascertained by calculating the difference between the intensity of solar radiation and the radiation through UV filtering goggles.

The dye displayed a distinct absorbance peak in visible light at a wavelength of 663 nm, where absorbance was at a maximum. This peak was used to monitor dye concentration in the solution over time. From the absorbance spectra corresponding to dye degradation, it was indisputable that the presence of titanium dioxide nanocatalysts resulted in a linear increase of the percentage of dye degradation with time, reflected by the decrease in absorbance. This finding indicated that when the time of irradiation was prolonged, the percent degradation increased and reached a maximum after 90 min of solar irradiation. When TiO_2_ nanocatalysts, dispersed in the dye solution, were irradiated with solar radiation, photo-generated charge carriers induced redox reactions on the surface of the photocatalyst. Essentially, any contaminant adsorbed onto the photocatalyst surface, by virtue of electron-hole pair generation, will undergo either reduction or oxidation, respectively. Generally, the TiO_2_ photocatalyst surface behaves as an active center in photocatalysis, either through the generation of OH radicals (by oxidation of OH^−^ anions) or by the generation of O_2_^−^ radicals (via the reduction of O_2_ molecules) [40]. Subsequently, these photo-generated radicals and anions react with the adsorbed organic contaminants to degrade or mineralize them into less harmful by-products, such as CO_2_ and H_2_O (Scheme 1).

**Figure 12:**
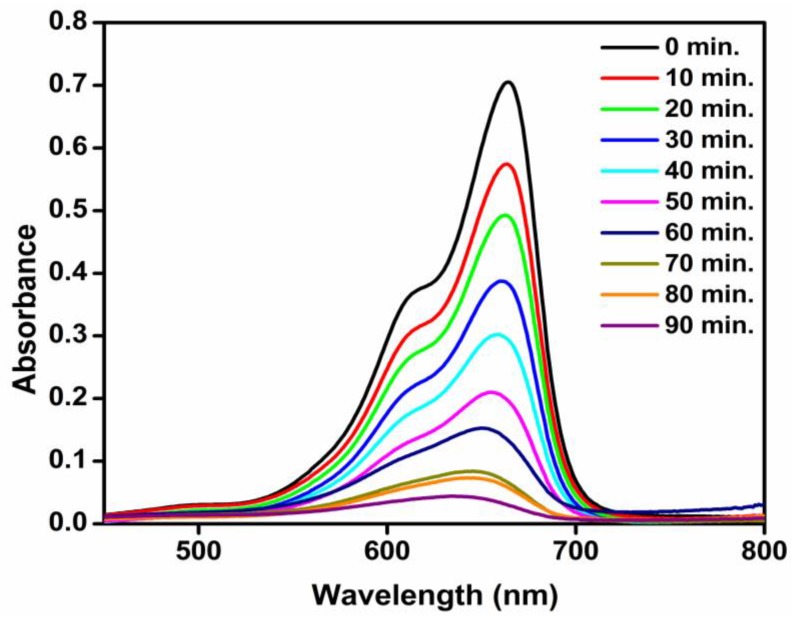
UV-visible spectra of methylene blue (MB) over various time intervals, in the presence of TiO_2_ nanocatalysts.

As the irradiation time lengthened, absorption of increasingly more light energy impinging on the catalyst surface occurred. This led to the increased formation of photo-excited species, and consequently, enhanced photocatalytic activity. From this study, it was observed that the photocatalytic dye degradation process was enhanced by lengthening exposure time (Figure 12). The Beer-Lambert-Bouguer law—relating the attenuation of light to the properties of the material through which the light is travelling—was used to determine molar concentrations of the degraded dyes.

No degradation of dye was discerned in the absence of solar light (supplementary information; Figure S2). The small decrease in absorbance observed was due to the insignificant adsorption of dye molecules on the catalyst surface. We have conducted a control experiment with MB on its own, as shown in supplementary information Figure S4. From this data, it is clear that TiO_2_ was activated by solar light and was responsible for dye degradation. Moreover, photocatalytic degradation of dye was confirmed by the supplementary information (Figure S5, i.e., in the absence of sunlight, there is no degradation observed, confirming the major role of sunlight in the activation of the TiO_2_ nanocatalyst). From Figures S4 and S5, it was confirmed that dye discoloration was entirely due to photocatalytic degradation, not adsorption.

#### 3.10.1. Effect of Catalyst Concentration on Photocatalytic Activity of TiO_2_ Nanoparticles

**Figure 13:**
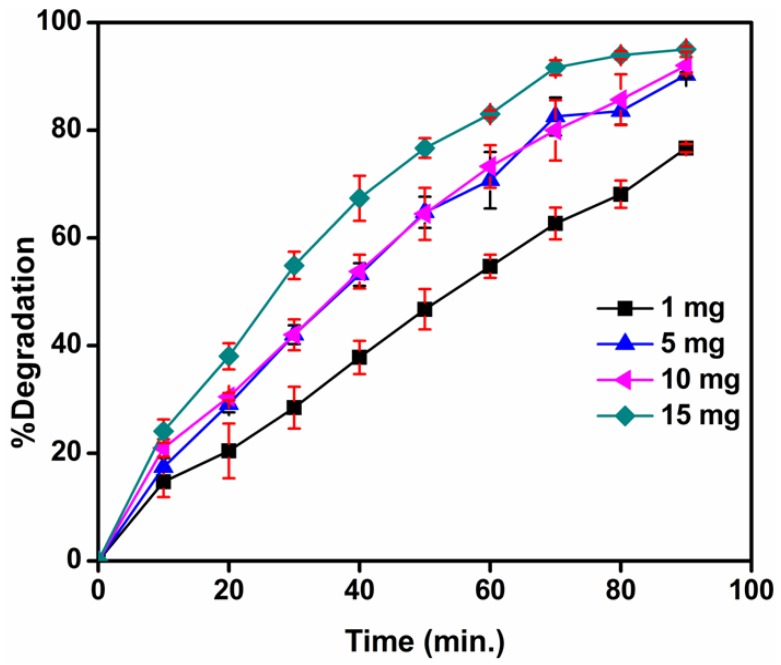
UV-visible spectra of MB dye (10^−5^ M) with different amounts of catalyst loading. Data obtained by degradation versus time are average values of three independent experiments (reported values = mean ± S.D.)

The effect of catalyst concentration on photocatalytic activity was tested by loading 1 to 15 mg/50 mL of TiO_2_ nanocatalyst in methylene blue solution. The photocatalytic degradation of methylene blue was highly influenced by the level of catalyst loading, as evident in Figure 13 which shows that the percent degradation of the dye increased with the amount (from 1 to 15 mg/50 mL) of TiO_2_ nanocatalyst loading and remained virtually constant above a certain level. This is because as the amount of catalyst increased, a greater number of active sites on the photocatalyst surface became available. Consequently, more OH radicals were produced, which facilitated their participation in the dye degradation process. However, beyond a certain limiting value of catalyst loading, the solution appeared turbid. As a result, the passage of solar radiation into the reaction mixture (required for the reaction to proceed) was obstructed, and thus, the percent degradation of the dye decreased or remained constant [41].

#### 3.10.2. Effect of pH on Photocatalytic Activity of TiO_2_ Nanoparticles

**Figure 14:**
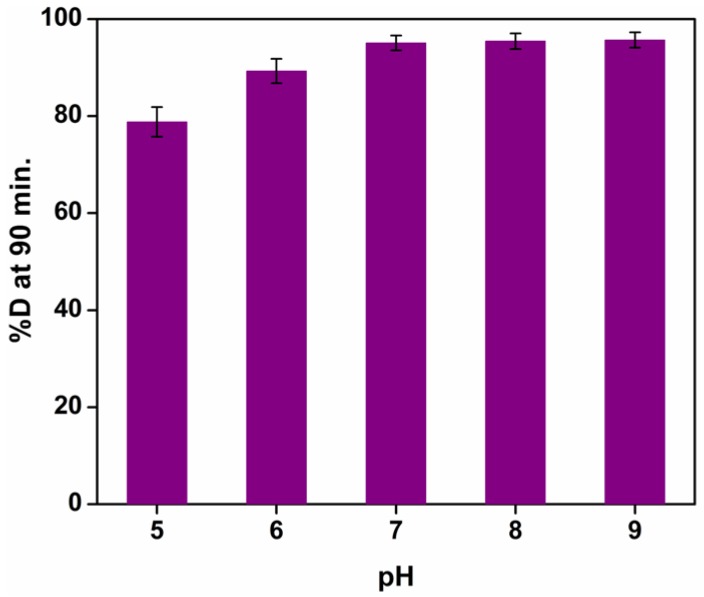
The photocatalytic degradation of MB dye (10^−5^ M) after treatment with photocatalyst TiO_2_ (0.2 g/L) in solar light under different pH conditions (reported values are for three independent experiments; average value = mean ± S.D; *n* = 3).

The role of pH on the rate of photocatalytic degradation was studied over the 4–9 pH range and the results are illustrated in Figure 14. It was observed that the percent degradation increased with a rising pH, exhibiting a maximum between the pH 7–9 ranges. This pH variation may bring about changes to the surface charge on the TiO_2_ nanoparticles and vary the potential associated with catalytic reactions. With the variation of potential, the extent of dye adsorption on the catalyst surface also fluctuates, culminating in the alteration of reaction velocity. Furthermore, under alkaline conditions, the surface of TiO_2_ could acquire a negative charge. Since methylene blue is a cationic dye and the surface of TiO_2_ nanoparticles in alkaline media attains a negative charge, the latter can be easily adsorbed onto the catalyst surface. This may lead to enhanced photocatalytic dye degradation under basic conditions [42]. The surface of TiO_2_ nanoparticles, in acidic or alkaline circumstances, can be protonated or deprotonated, respectively, according to the following reactions:

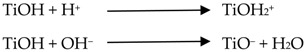


#### 3.10.3. Effect of Calcination Temperature on Photocatalytic Activity of TiO_2_ Nanoparticles

**Figure 15:**
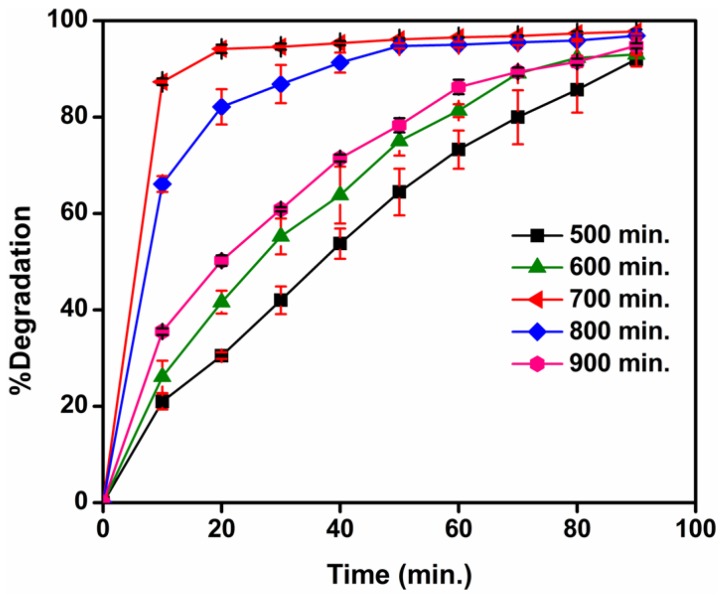
The photocatalytic degradation of MB dye (10^−5^ M) after treatment with photocatalyst TiO_2_ (0.2 g/L) calcined at different temperatures under solar light (Values were reported as mean ± S.D; *n* = 3).

The effect of calcination temperatures of the TiO_2_ nanocatalyst on photocatalytic degradation is depicted in Figure 15. With increasing calcination temperatures of the TiO_2_ nanocatalyst, the percentage degradation was found to have risen and attained a maximum of 96.85% at 700 °C. It then decreased gradually at temperatures between 800 °C and 900 °C. This variation can be expected, given that an increase in calcination temperature of the TiO_2_ generated an anatase phase possessing good crystal characteristics—an essential criterion for photocatalytic degradation. It is also apparent from the UV data (Table 1) that the band gap increased with rising calcination temperatures, from 500 ^o^C to 800 °C. Hence, the rate of electron-hole recombination decreased, and consequently, photocatalytic degradation increased. The decline in photocatalytic degradation at 800 °C was mainly ascribed to the formation of the rutile phase, which existed as a mixture of both the anatase and rutile forms. Complete formation of the rutile phase transpired at 900 °C, and thus site activity fell and degradation decreased once again.

It is clear that the photocatalytic ability of titanium dioxide NPs is largely dependent on its crystalline form. Thermodynamically, the efficiency corresponding to photo-oxidation by the anatase and rutile phases should be similar. The surface recombination of photo-excited electrons and positive holes was higher in rutile than in anatase, which has a greater free (electron)-carrier mobility. Hence, a range of photoactivities was observed for each crystal form. This showed that variables such as crystal type and particulate sizes, as well as various synthesis routes (temperature, heating time etc.) largely determine its photocatalytic activity [40].

## 4. Conclusions

Green nanotechnology has, in recent years, been accorded increasing importance for two reasons, namely, its contribution towards the elimination of harmful reagents and its ability to facilitate the synthesis of valuable products in a cost-effective manner. The green synthesis of TiO_2_ NPs involves more compatible, eco-friendly, low cost and less time-consuming processes compared to other synthetic methods such as the sol-gel technique, which has been widely used to achieve the same ends. In the present study, titanium dioxide nanoparticles were produced using a natural hydrocolloid, gum Kondagogu (*Cochlospermum gossypium*). The crystallinity and high purity of the synthesized TiO_2_ nanoparticles in the anatase form were unambiguously confirmed by the presence of sharp peaks (and the absence of unidentified peaks) in the X-Ray Diffraction patterns, Raman spectroscopic results and TEM images obtained. The absence of the original gum residue on the nanoparticles after calcination for 4 h at 500 °C was confirmed by FTIR, EDX, BET and TGA. The nanoparticles created had a mean particle size of approximately 11 nm, a value which was determined by three independent techniques: Scherrer’s formula from XRD (12.58 nm); TEM (8–13 nm) and CPS (11.2 ± 0.2 nm), the figures in parentheses being the respective particle sizes. The photocatalytic activity of green-synthesized titanium dioxide nanoparticles was evaluated by adopting methylene blue dye as a model system. It is apparent that the photocatalytic effectiveness of titanium dioxide nanoparticles is largely dependent on various factors including its crystalline form, exposure time, extent of catalyst loading and solution pH conditions. The present study has demonstrated that titanium dioxide nanoparticles (synthesized through a green route by the use of natural, renewable and eco-friendly materials) exhibit excellent photocatalytic activity towards organic dye degradation. This system can be employed in water purification and dye effluent treatment.

## Figures and Tables

**Table 1:** Band gap of TiO2 nanoparticles calcined at different temperatures.

Calcination Temperatures of TiO_2_ (°C)	Band Gap (eV)
500	3.13
600	3.15
700	3.16
800	3.18
900	3

## Supplementary Materials

The following are available online at http://www.mdpi.com/2079-4991/8/12/1002/s1, Figure S1: The intensity of solar light measured throughout the experiment (range 100,000 Lux); Figure S2: The % of UV intensity in solar radiation at various intervals of time; Figure S3: The solar UV intensity measurement using Lux meter and UV filtering goggles; Figure S4: UV-visible spectra of MB over various time intervals after treatment with TiO2 nanocatalyst under dark condition; Figure S5: UV-visible spectra of MB over various time intervals in the absence of TiO2 nanocatalyst.
