# Supplementary material for: Green Synthesis of High Temperature Stable Anatase Titanium Dioxide Nanoparticles Using Gum Kondagogu: Characterization and Solar Driven Photocatalytic Degradation of Organic Dye
Source: Nanomaterials (Basel). 2018 Dec 4;8(12):1002. doi: 10.3390/nano8121002 (PMC6316888; doi:10.3390/nano8121002)
Supplement: Supplementary file 1 [file nanomaterials-08-01002-s001.zip › nanomaterials-383705-SI.pdf]

nanomaterials

Article

Green Synthesis of High Temperature Stable Anatase Titanium Dioxide Nanoparticles Using Gum Kondagogu: Characterization and Solar Driven Photocatalytic Degradation of Organic Dye

Saranya K.S. [1], Vinod Vellora Thekkae Padil [2,*], Chandra Senan [3], Rajendra Pilankatta [4], Saranya K. [1], Bini George [1,*], Stanisław Wacławek [2] and Miroslav Černík [2,*]

[1] Department of Chemistry, School of Physical Sciences, Central University of Kerala, Kerala 671316, India; sharanyacks@gmail.com (S.K.S.); kunjumonsaranya916@gmail.com (S.K.)
[2] Institute for Nanomaterials, Advanced Technologies and Innovation (CXI),Technical University of Liberec (TUL), Studentská 1402/2, 46117 Liberec 1, Czech Republic; stanislaw.waclawek@tul.cz
[3] Centre for Water Soluble Polymers, Applied Science, Faculty of Arts, Science and Technology, Wrexham Glyndwr University, Wrexham LL11 2AW, Wales, U.K.; c.senan@glyndwr.ac.uk
[4] Department of Biochemistry and Molecular Biology, School of Biological Sciences, Central, University of Kerala, Kerala 671316, India; praj74@gmail.com
* Correspondence: vinod.padil@tul.cz (V.V.T. P); binigeorgek@gmail.com (B.G.); miroslav.cernik@tul.cz (M.Č.); Tel.: +420-723-372-911 (V.V.T. P& M.Č.)

Received: 28 October 2018; Accepted: 01 December 2018; Published: December 2018

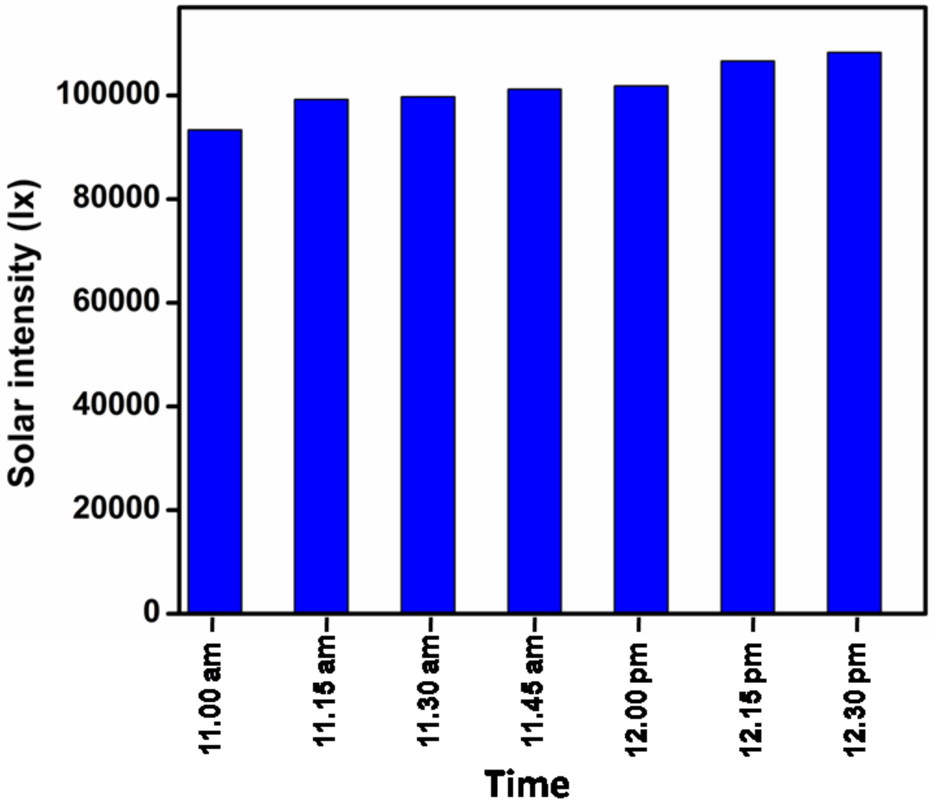

Figure S1. The intensity of solar light measured throughout the experiment (range 100,000 Lux).

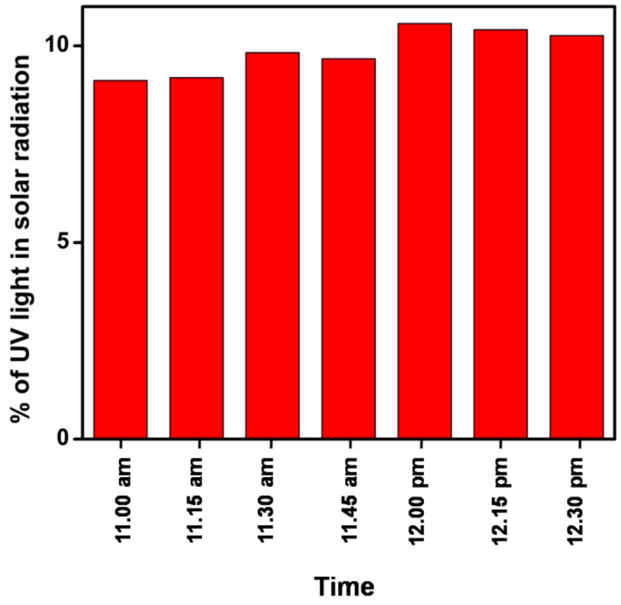

Figure S2. The % of UV intensity in solar radiation at various intervals of time.

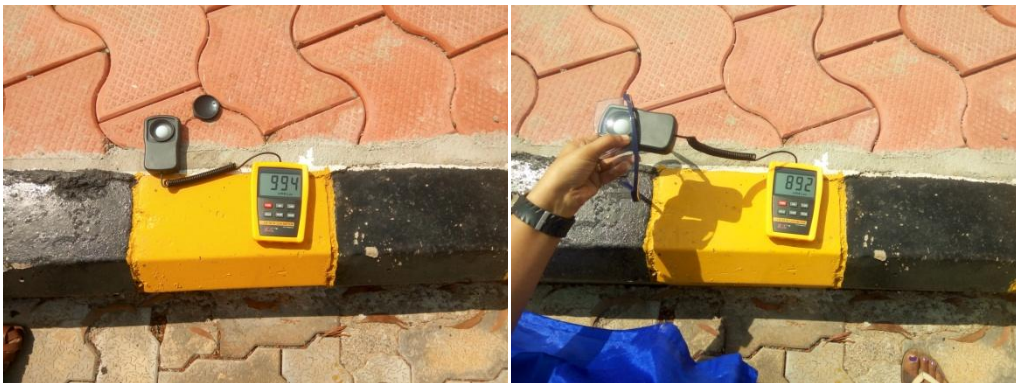

Figure S3. The solar UV intensity measurement using Lux meter and UV filtering goggles.

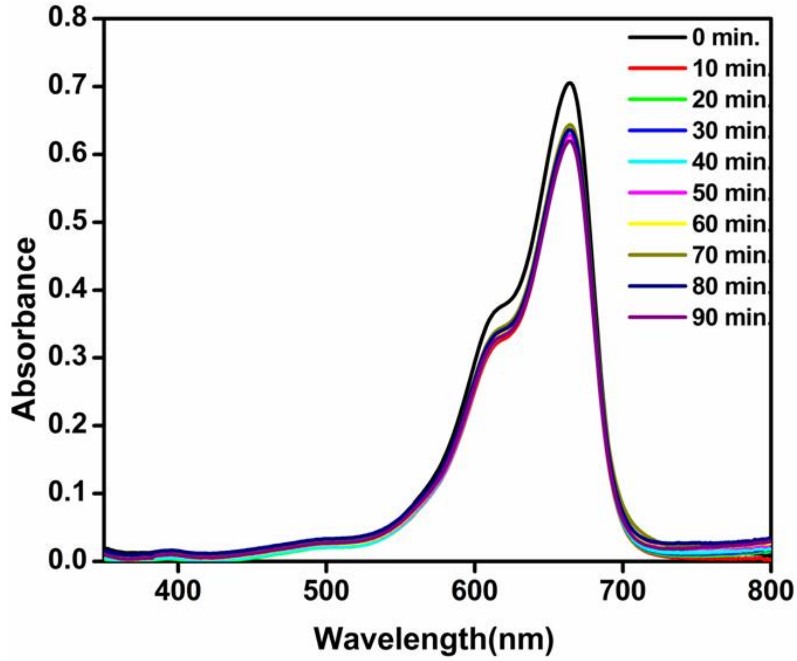

Figure S4. UV-visible spectra of MB over various time intervals after treatment with TiO_2 nanocatalyst under dark condition.

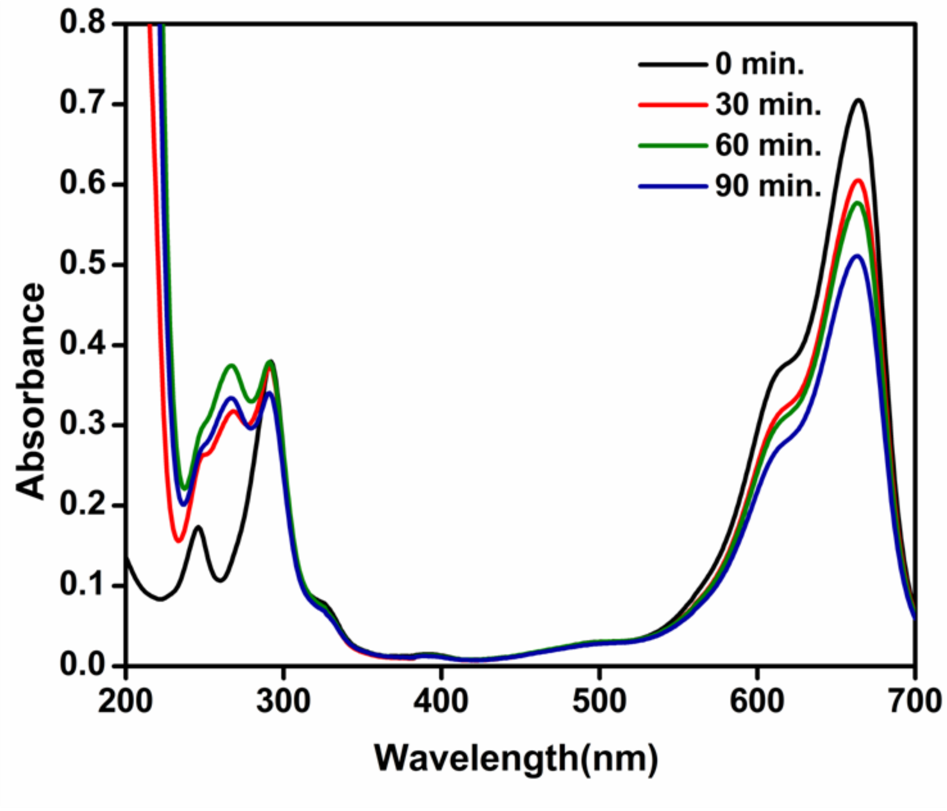

Figure S5. UV-visible spectra of MB over various time intervals in the absence of TiO_2 nanocatalyst.
